# Comparison between the Transcriptomes of ‘KDML105’ Rice and a Salt-Tolerant Chromosome Segment Substitution Line

**DOI:** 10.3390/genes10100742

**Published:** 2019-09-24

**Authors:** Nopphawitchayaphong Khrueasan, Panita Chutimanukul, Kitiporn Plaimas, Teerapong Buaboocha, Meechai Siangliw, Theerayut Toojinda, Luca Comai, Supachitra Chadchawan

**Affiliations:** 1 Center of Excellence in Environment and Plant Physiology, Department of Botany, Faculty of Science, Chulalongkorn University, Bangkok 10300, Thailand; sharesci@gmail.com (N.K.); priggerr@gmail.com (P.C.); 2 Advanced Virtual and Intelligent Computing (AVIC) Center, Department of Mathematics and Computer Science, Faculty of Science, Chulalongkorn University, Bangkok 10330, Thailand; 3 Molecular Crop Research Unit, Department of Biochemistry, Faculty of Science, Chulalongkorn University, Bangkok 10330, Thailand; teerapong.b@chula.ac.th; 4 Omics Science and Bioinformatics Center, Faculty of Science, Chulalongkorn University, Bangkok 10300, Thailand; 5 Rice Gene Discovery Unit, National Center for Genetic Engineering and Biotechnology, Kasetsart University, Kamphangsaen Campus, Nakhonpathom 73140, Thailandtoojindatheerayut@gmail.com (T.T.); 6 Department of Plant Biology, UC Davis Genome Center, UC Davis, Davis, CA 95616, USA

**Keywords:** phototropin, pyruvate dehydrogenase kinase, two‐component signaling system

## Abstract

‘KDML105’ rice, known as jasmine rice, is grown in northeast Thailand. The soil there has high salinity, which leads to low productivity. Chromosome substitution lines (CSSLs) with the ‘KDML105’ rice genetic background were evaluated for salt tolerance. CSSL18 showed the highest salt tolerance among the four lines tested. Based on a comparison between the CSSL18 and ‘KDML105’ transcriptomes, more than 27,000 genes were mapped onto the rice genome. Gene ontology enrichment of the significantly differentially expressed genes (DEGs) revealed that different mechanisms were involved in the salt stress responses between these lines. Biological process and molecular function enrichment analysis of the DEGs from both lines revealed differences in the two-component signal transduction system, involving *LOC_Os04g23890*, which encodes phototropin 2 (*PHOT2*), and *LOC_Os07g44330*, which encodes pyruvate dehydrogenase kinase (PDK), the enzyme that inhibits pyruvate dehydrogenase in respiration. *OsPHOT2* expression was maintained in CSSL18 under salt stress, whereas it was significantly decreased in ‘KDML105’, suggesting OsPHOT2 signaling may be involved in salt tolerance in CSSL18. *PDK* expression was induced only in ‘KDML105’. These results suggested respiration was more inhibited in ‘KDML105’ than in CSSL18, and this may contribute to the higher salt susceptibility of ‘KDML105’ rice. Moreover, the DEGs between ‘KDML105’ and CSSL18 revealed the enrichment in transcription factors and signaling proteins located on salt-tolerant quantitative trait loci (QTLs) on chromosome 1. Two of them, *OsIRO2* and *OsMSR2*, showed the potential to be involved in salt stress response, especially, *OsMSR2*, whose orthologous genes in *Arabidopsis* had the potential role in photosynthesis adaptation under salt stress.

## 1. Introduction

Salt stress is one of the most serious environmental stresses limiting crop growth and productivity worldwide. Therefore, the development of salt-tolerant rice cultivars is an important aim of rice breeding programs, especially in Thailand, other parts of Asia, and Africa. Salt tolerance in rice is a complex trait that is regulated by many genes and strongly influenced by the environment [1,2]. A number of quantitative trait loci (QTLs) for drought tolerance have been detected in rice, and many genes that respond to both drought and salt stresses have been identified [2]. Indeed, many plant genes are activated by both drought and salinity conditions [3,4].

‘KDML105’ rice, known as jasmine rice worldwide, is a popular rice cultivar from Thailand. Near-isogenic lines (NILs) with the ‘KDML105’ rice genetic background were developed from chromosome segment substitution lines (CSSLs) that carry QTLs for drought tolerance [5]. These NILs were derived by crossing ‘KDML105’ with doubled haploid line 212 (DH212) as a donor. DH212 was developed by crossing rice lines CT9993 and IR62266 to create a source of drought resistance genes for use in rice breeding programs [6]. Some of these NILs contained a QTL related to drought tolerance in the region between markers RM212 and RM3362 and showed good salt tolerance ability under salt stress. Therefore, we selected CSSLs that had the same sequence as DH212 between these markers and investigated their salt tolerance. The CSSL that showed the best performance under salt stress was selected and its transcriptome sequence was compared with the transcriptome of parental ‘KDML105’ rice.

Comparing the transcriptomes of different rice genotypes is useful for discovering genes under selected conditions and for clarifying the roles of biological pathways and mechanisms under different conditions. However, for completely divergent rice genotypes with contrasting phenotypes, transcriptome analysis can be challenging because of high genetic background noise, which makes it difficult to focus on specific genes; for example, salt-responsive genes. Therefore, comparing rice NILs with the recurrent parent, like CSSL, decreases the genetic background noise in the transcriptomic analysis because the NILs carry only small segments of genomic sequences from a known salt-tolerant donor. In this study, the transcriptomes of a salt-tolerant NIL and its genetic background parent were analyzed under salt stress to detect changes in gene expression. The objective was to understand the genetic mechanisms that are active in rice under salt stress.

## 2. Materials and Methods

### 2.1. Plant Materials

**Figure 1:**
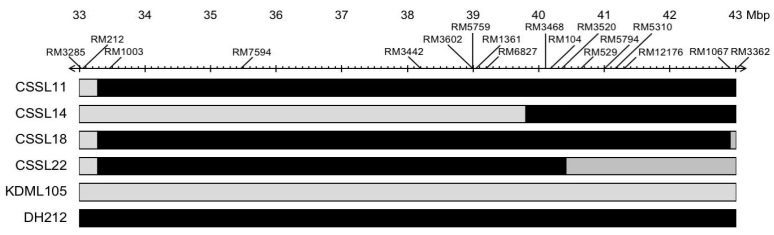
Simple sequence repeat (SSR) markers in the salt-tolerant quantitative trait locus (QTL) region [5] of the rice lines used in this study. Black indicates the genomic DNA from DH212, the donor parent, and the gray indicates the genomic DNA from ‘KDML105’, the recurrent parent. The chromosome substitution lines (CSSLs) were checked with SSR markers in other regions of the genome and >95% were in the ‘KDML105’ genome.

CSSLs with the ‘KDML105’ genetic background were generated at the Rice Gene Discovery Unit, National Center for Genetic Engineering and Biotechnology, Thailand [5]. Four CSSLs, CSSL11, CSSL14, CSSL18, and CSSL22, were selected and their leaf water potential under salt stress was evaluated. These CSSLs are a BC_5_F_2_ NIL with a few chromosome fragments introgressed from the donor parent’s, DH212, salt-tolerant line into the background of the recurrent parent ‘KDML105’ (Figure 1).

### 2.2. Physiological Trait Evaluation

The experiments were conducted in a greenhouse at the Department of Botany, Faculty of Science, Chulalongkorn University, Bangkok, Thailand. Rice seeds were germinated for 5 days in 150 mL plastic cups filled with distilled water, then transferred to a plastic pot containing clay soil following [5] under natural conditions in a greenhouse. The average temperature was 29.6 °C, humidity was 65–70%, and the day length was 12 h/day. For the salt stress treatment, 75 mM or 150 mM NaCl (Dominion Salt, New Zealand) was added to the nutrient solution as described below.

To evaluate leaf water potential, the four selected CSSLs (CSSL11, CSSL14, CSSL18 and CSSL22) and the parental lines, ‘KDML105’ and DH212, as well as the salt-tolerant standard, ‘Pokkali’ rice, and salt-susceptible standard, IR29, were grown in soil with full irrigation for 21 days. Then, the seedlings were separated into three groups and a completely randomized design was used to evaluate the response. One group was maintained under the normal conditions. The second and third groups were treated by adding 75 mM or 150 nM NaCl to the nutrient solution, respectively. Leaf water potential was determined by selecting the youngest fully expanded leaf of each seedling at 0, 4, 8, and 12 days after treatment at midday (11:00–13:00) with the Plant Water Status Console (model 3005) (Soilmoisture Equipment Corp., USA).

To compare the salt injury scores, fresh weights, and relative growth rates among the seedlings of all lines, the seedlings were grown in clay soil as described above. Salt stress was induced by adding 75 mM NaCl to the nutrient solution. Salt injury scores were determined following the standard evaluation system for salinity tolerance at the seedling stage in rice [7] daily after NaCl treatment for 6 days. Root and shoot fresh weights were determined after 14 days of salt stress and relative growth rate (RGR) was calculated as follows:

RGR = (ln W_2_ − ln W_1_)/(t_2_ − t_1_), (1)

where: W_1_ = fresh weight at the first time point, W_2_ = fresh weight at the second time point, t_1_ = first time point, t_2_ = second time point.

The experiment was performed using a completely randomized design with three replications. Each replication consisted of three samples.

### 2.3. RNA Extraction and Illumina Sequencing

Leaf tissues were collected for each genotype 0 and 2 days after the salt stress treatment. Plant RNA Purification Reagent (Thermo Fisher Scientific, Waltham, MA, USA) was used to extract total RNA. DNA was removed from the total RNA using DNase I, amplification grade (Thermo Fisher Scientific, Waltham, MA, USA). A KAPA Stranded mRNA-Seq Kit (Kapa Biosystems, Wilmington, MA, USA) was used to construct the RNA-Seq libraries. All the steps were quantified using a Qubit RNA HS Assay Kit or Qubit dsDNA HS Assay Kit (Thermo Fisher Scientific, Waltham, MA, USA). Three libraries for each of the groups were loaded into the Illumina HiSeq 3000 sequencing system (Illumina, San Diego, CA, USA) and sequenced using the 50SE (single end) strategy.

### 2.4. Transcriptome Data Analysis

The short-sequence reads from the Illumina sequencing system were grouped into the categories using the pipeline developed by Missirian et al. [8]. The sequence reads were aligned and mapped to the rice genome database (RGAP 6.0) using Bowtie 2 [9] and TopHat [10].

### 2.5. Identification of Differentially Expressed Genes

Differentially expressed genes (DEGs) were identified using the DESeq package (version 1.24.0) [11]. Transcripts with a log2 fold change ≥1 (upregulated genes) and ≤1 (downregulated genes), and DEGs with a *p*-value cut off ≤0.05 adjusted with the Benjamini–Hochberg procedure were considered significant.

### 2.6. Gene Ontology Enrichment and Pathway Analysis

Gene ontology (GO) enrichment was performed for the DEGs using the BiNGO plug-in (version 3.0.3) [12] on the Cytoscape platform (version 3.4.0; http://www.cytoscape.org/). The GO biological process and molecular function annotations for rice were used for the enrichment analysis. A *p*-value cut off ≤0.05 was considered significant and a hypergeometric test was applied to identify enriched GO terms in BiNGO. The pathway analysis of the DEGs was performed using MapMan (version 3.5.1; http://mapman.gabipd.org/web/guest) [13] with a *p*-value cut off ≤0.05.

### 2.7. Gene Expression Analysis

A total of 1 μg of DNase-treated total RNA was converted into complementary DNA (cDNA) using an iScript cDNA Synthesis Kit (Bio-rad, Hercules, CA, USA). The total volume of the RT-qPCR reaction was 10 μL, consisting of 5 μL SYBR green fluorophore (Biotech Rabbit, Hennigsdorf, Germany), 0.25 μL of 2.5 mM forward and reverse primers, 1 μL cDNA, and 3.50 μL water. The PCRs were run on a Bio-Rad CFX Real-Time Thermal Cycler (Bio-rad, Hercules, CA, USA). Three technical replicates (triplicates) were performed for each biological replicate. The rice elongation factor 1α gene (*EF1a*; *LOC_Os03g08030*) was used as a reference. Relative expression levels were calculated by the 2^−ΔΔCT^ method following [14].

### 2.8. Determination of the Involvement of the Predicted Genes in Salt Stress Response by Using Homolog Arabidopsis Mutant Lines

*Arabidopsis* mutants were ordered from the Arabidopsis Biological Resource Center. The homozygous T-DNA insertion mutant lines [15] were selected for further characterization. *Arabidopsis* seeds were germinated in 1/2 MS media [16] after stratification at 4 °C for 2 days. Five-day-old seedlings were transplanted to grow on materials composed of peat (Klasmann-Deilmann, Geeste, Germany): perlite (RHP, ′s-Gravenzande, Netherlands): vermiculite (RHP, ′s-Gravenzande, Netherlands) at the ratio 3:1:1. Plants were grown in a growth room at 25 °C with a light intensity 50 µmole m^−1^ s^−1^ at 16 hours a day. The stress response experiment was done with 15-day-old seedlings in complete randomized design (CRD) with four replicates. The plants were separated into two treatments for normal growth (control) and salt stress. For salt stress treatment, seedlings were treated with 250 mM NaCl solution, while filtered water was used in the control set. After treatment for 7 days, the quantum yield efficiency of the photosystem II (*F_v_*/*F_m_*) and photosynthetic performance index (*P_i_*) were measured on 40 min dark adapted leaves with a Pocket PEA rapid screening chlorophyll fluorimeter (Hansatech Instruments, Norfolk, UK). Then, fresh and dry weights of above ground tissues were determined. RGR of all lines were also determined by collecting the above-grown tissues at the beginning and end (7 days after treatment) of the experiment and calculated with the equation shown in (1).

## 3. Results

### 3.1. CSSL18 Has Higher Salt Tolerance than ‘KDML105’

**Figure 2:**
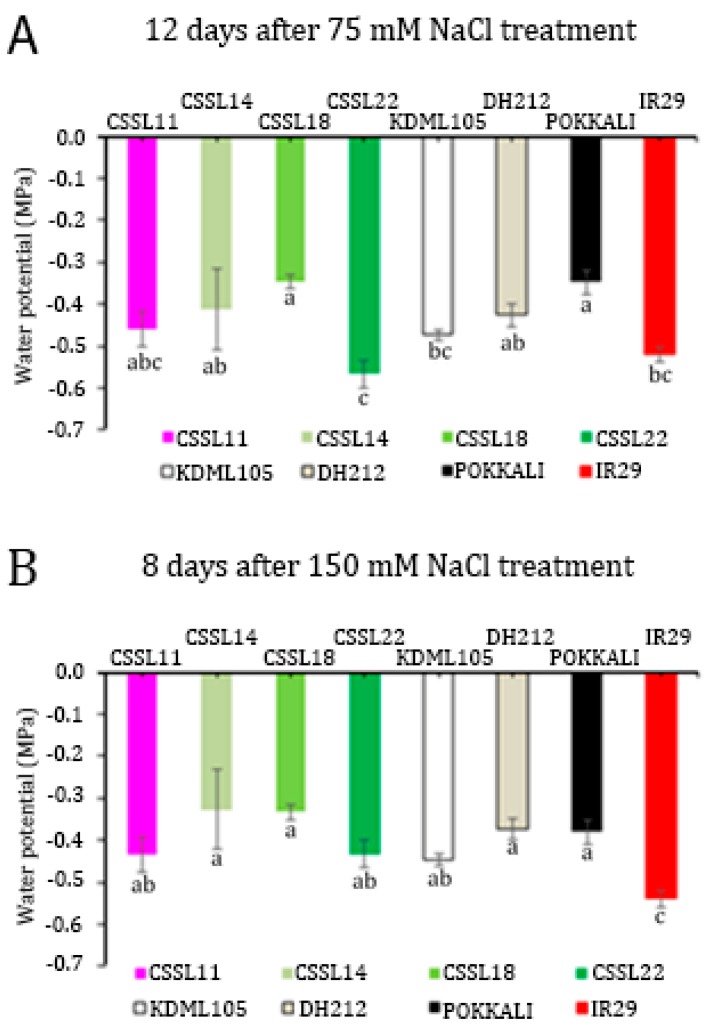
Leaf water potential of four CSSLs, parental lines (‘KDML105’ and DH212), and salt-tolerant (‘Pokkali’) and salt-susceptible (IR29) lines treated with 75 mM NaCl for 12 days (**A**) and 150 mM NaCl for 8 days (**B**). The different lower case letters show the significant difference of means, *p* < 0.05.

When the seedlings were grown under normal conditions, the leaf water potential of all the lines was similar at about −0.25 mPa (Figure S1A). When the seedlings were subjected to salt stress, the leaf water potential of the different lines declined at different rates, resulting in significantly different leaf water potentials at certain time points (Figure S1). The leaf water potentials of the tested lines showed significant differences after 12 days of 75 mM NaCl treatment, (Figure 2A) and after only 8 days of 150 mM NaCl treatment (Figure 2B). ‘Pokkali’ and CSSL18 had the highest leaf water potential under both salt stress conditions, including being higher than the ‘KDML105’ leaf water potential, when grown under the same conditions.

**Figure 3:**
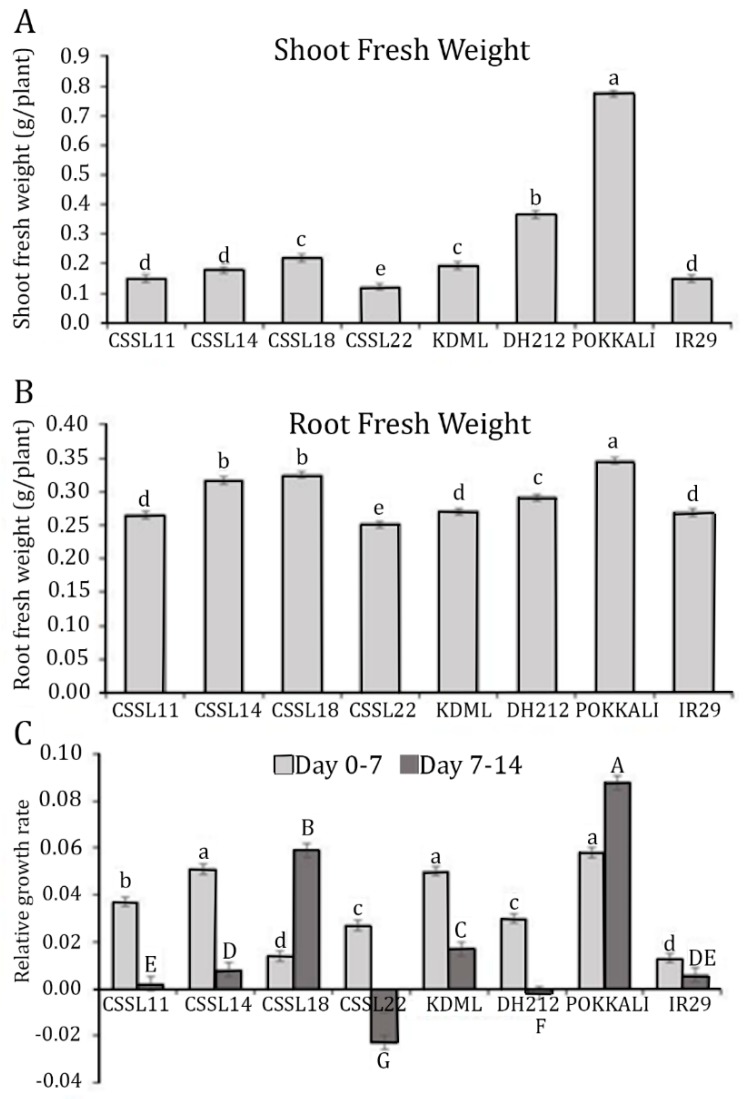
Growth of CSSLs, parental lines (‘KDML105’ and DH212), and salt-tolerant (‘Pokkali’) and salt-susceptible (IR29) lines, determined by shoot fresh weight (**A**), root fresh weight (**B**), and relative growth rate during 0–7 days and 7–14 days of salt stress (**C**). The different lower case letters or capital letters show the significant difference of means, *p* < 0.05.

‘Pokkali’ had the highest shoot and root fresh weights when the seedlings were treated with 150 mM NaCl for 14 days. CSSL18 and ‘KDML105’ had similar shoot fresh weights, which were significantly lower than the shoot fresh weight of DH212. The other CSSLs and IR29 had shoot fresh weights that were lower than those of both parents (Figure 3A). However, CSSL14 and CSSL18 had higher root fresh weights than both parents (Figure 3B).

Relative growth rate (RGR), measured on the dry weight of the above ground tissues, was determined at two time periods, zero to seven days and 7–14 days after salt stress. Among the CSSLs, CSSL14 had the highest RGR at zero to seven days after salt stress, which was similar to the RGRs of ‘KDML105’ and ‘Pokkali’. IR29 had the lowest RGR during this period. At 7–14 days after salt stress, ‘Pokkali’ had the significantly highest RGR, and among the CSSLs, CSSL18 had the highest RGR, which was significantly higher than the RGR of ‘KDML105’ during this period (Figure 3C). CSSL22 and DH212 had negative RGRs at 7–14 days after salt stress, suggesting abscission of leaf tissues had occurred.

Overall, CSSL18 showed the best salt-tolerant phenotype among the CSSLs. Therefore, we selected CSSL18 for comparison with ‘KDML105’ in the transcriptome analysis.

### 3.2. Comparison of the Transcriptomes of CSSL18 and ‘KDML105’ Rice Seedlings

**Figure 4:**
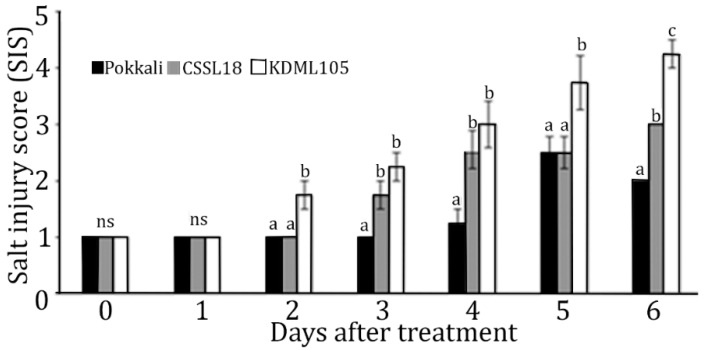
Salt injury scores for CSSL18, ‘KDML105’, and ‘Pokkali’ rice seedlings treated with 75 mM NaCl as a check for the transcriptomic study of CSSL18 and ‘KDML105’. The different lower case letters show the significant difference of means, *p* < 0.05.

To prepare the leaf tissues for the transcriptomic study, CSSL18, ‘KDML105’, and ‘Pokkali’ rice seedlings were grown for 21 days and subjected to salt stress by adding 75 mM NaCl into the nutrient solution. The salt injury score (SIS) was collected daily for 6 days after salt stress. The control set had an SIS of one, suggesting the growing system was proper. The SIS of ‘KDML105’ was higher than the SIS of the other two lines after 2 days of salt stress, and was the significantly highest SIS after 6 days under salt stress (Figure 4). Therefore, we collected leaf tissues of CSSL18 and ‘KDML105’ after salt stress treatment for zero and two days for transcriptome analysis to investigate early changes in gene expression that may affect the salt-tolerant phenotype of CSSL18.

Clean cDNA reads from the ‘KDML105’ transcriptome were mapped onto the rice reference genome [17]. They identified 27,610 loci. After two days of salt stress, 1167 genes were significantly differentially expressed compared with their expression at zero days; 584 genes were upregulated and 583 genes were downregulated (Table S1).

The clean reads from the CSSL18 transcriptome mapped to 28,064 loci on the rice reference genome. After two days of salt stress, 920 genes were significantly differentially expressed compared with their expression at zero days; 473 genes were upregulated and 447 genes were downregulated (Table S2).

**Figure 5:**
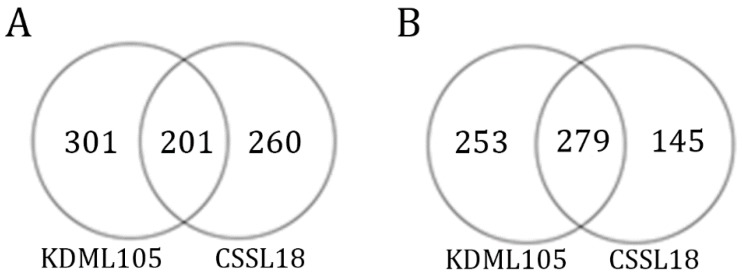
Numbers of significantly differentially expressed genes in response to salt stress. (**A**) Numbers of upregulated genes; (**B**) numbers of downregulated genes.

Among the DEGs, 480 genes in both lines matched the criterion of having a log2 fold change >1 or <−1; 201 genes were upregulated and 279 genes were downregulated (Table S3). The number of genes that were differentially expressed only in ‘KDML105’ or CSSL18 are shown in Figure 5 and are listed in Tables S4 and S5, respectively.

### 3.3. GO Enrichment Analysis of the Significant DEGs Reveals Different Mechanisms Involved in Salt Stress Responses between ‘KDML105’ and CSSL18

Both upregulated and downregulated DEGs in ‘KDML10’ and CSSL18 were subjected to a GO enrichment analysis using the BiNGO plug-in (version 3.0.3) [12] on the Cytoscape platform (version 3.4.0; http://www.cytoscape.org/). Two of the main GO categories were selected, biological process and molecular function, to investigate differences in the mechanisms involved in the salt stress responses of ‘KDML105’ and CSSL18 at the early stage of stress.

**Figure 6:**
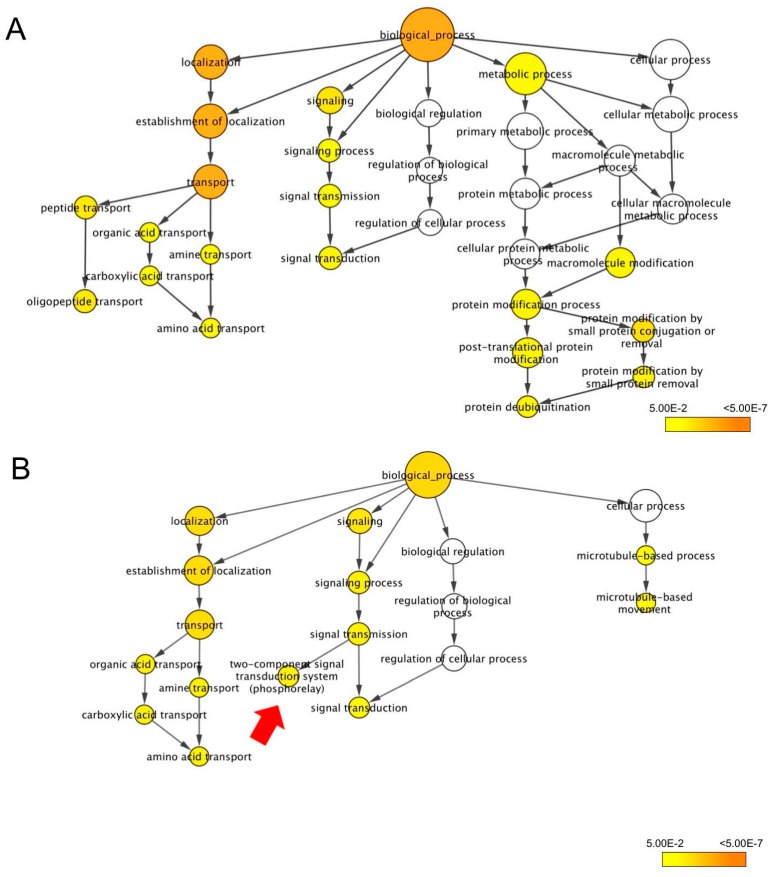
Biological process gene ontology (GO) terms enriched for the differentially expressed genes (DEGs) in ‘KDML105’ (**A**) and CSSL18 (**B**) by BiNGO. The red arrow indicates a term enriched only for CSSL18 DEGs.

Under the biological process, three main terms were found for the DEGs in both ‘KDML105’ and CSSL18, namely, localization, signaling, and the cellular process. The metabolic process was found only for ‘KDML105’ DEGs, and the protein metabolic process and molecular metabolic process were the main terms found under the cellular process for ‘KDML105’ DEGs. The microtubule process was the only enriched term found under the cellular process for the CSSL18 DEGs, and this term was not enriched for ‘KDML105’ DEGs (Figure 6).

Under localization, organic transport, carboxylic transport, and amine and amino acid transport were enriched for the DEGs in both ‘KDML105’ and CSSL18, whereas peptide and oligopeptide transport was significantly enriched only for ‘KDML105’ DEGs (Figure 6).

Interestingly, under signaling, the enriched terms for the DEGs in both lines were similar, except the two-component signal transduction system term was found only for CSSL18 DEGs (Figure 6B). Signaling processes are one of the early responses in plants facing stress, so the DEGs enriched in these terms may contribute to the salt stress response and salt tolerance ability of these lines.

**Figure 7:**
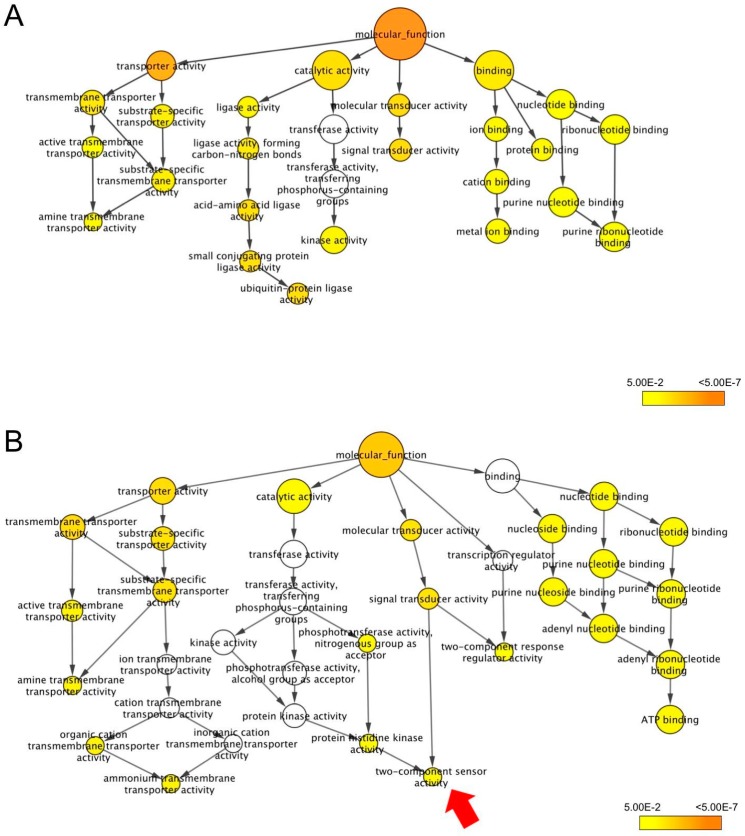
Molecular function GO terms enriched for the differentially expressed genes (DEGs) in ‘KDML105’ (**A**) and CSSL18 (**B**) by BiNGO. The red arrow indicates a term enriched only for CSSL18 DEGs.

Under the molecular function, transport activity was enriched in both lines (Figure 7), which is consistent with the enrichment of transport terms found under the biological process (Figure 6). Interestingly, the organic cation transmembrane transport activity and ammonium transmembrane transport activity were significantly enriched only for CSSL18 DEGs, including *LOC_Os01g42740* and *LOC_Os04g43070*, both of which encode an ammonium transporter protein. Under catalytic activity, ligase activity was the most enriched term for ‘KDML105’ DEGs (Figure 7A), whereas phosphotransferase activity and protein histidine kinase activity were enriched for CSSL18 DEGs. Two-component sensor activity (Figure 7B), which is related to molecular transducer activity, was enriched only in CSSL18 DEGs.

Based on the enriched terms under the two main GO categories, we focused on two genes, *LOC_Os04g23890* and *LOC_Os07g44330*, annotated as being involved in the two-component signaling system, which may contribute to the differences in the salt stress response between ‘KDML105’ and CSSL18. These genes were predicted to encode an ACG kinase and kinase protein, respectively. Information from the Rice Genome Annotation Project [17] indicates orthologous genes of *LOC_Os04g23890* encode phototropin 2 (PHOT2) and orthologous genes of *LOC_Os07g44330* encode pyruvate dehydrogenase kinase (PDK).

### 3.4. Differential Expression of LOC_Os04g23890 and LOC_Os07g44330 in Rice

**Figure 8:**
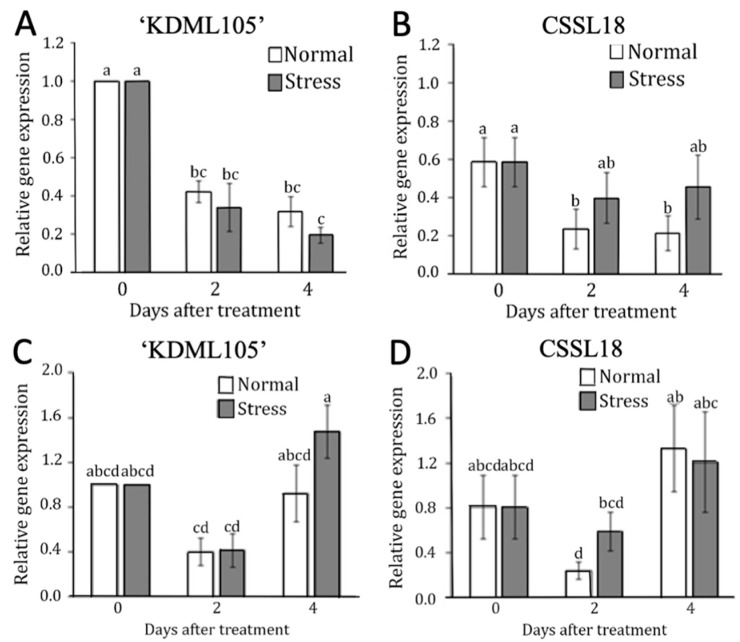
Relative expression of *OsPHOT2* (**A**,**B**) and *OsPDK* (**C**,**D**) in ‘KDML105’ (**A**,**C**) and CSSL18 (**B**,**D**) in ‘KDML105’ (**A**) and CSSL18 (**B**) under normal and salt stress conditions for 0, 2, and 4 days. The different lower case letters show the significant difference of means, *p* < 0.05.

We performed quantitative RT-PCRs to confirm the expression levels of *LOC_Os04g23890* (*OsPHOT2*) and *LOC_Os07g44330* (*OsPDK*) in ‘KDML105’ and CSSL18. The gene expression levels were compared using a reference expression in ‘KDML105’ under normal growth conditions. We found that *OsPHOT*2 expression in ‘KDML105’ was not affected by salt stress (Figure 8A), while *OsPHOT2* was induced in CSSL18 after two days of salt stress (Figure 8B). This was consistent with RNAseq data (Table S5).

For *OsPDK*, gene expression tended to increase in ‘KDML105’ after four days of salt stress (Figure 8C), but for CSSL 18, the salt-treated seedlings showed a similar level to the ones grown in the normal condition (Figure 8D). However, this gene tended to be induced in CSSL18 after two days of salt stress. This pattern of response was different from the RNASeq data, in which a slightly higher induction was found in ‘KDML105’ than in CSSL18 after two days of salt stress (Table S3). 

### 3.5. Identification of transcription factors using MapMan analysis

To identify salt tolerance involving transcription factors, transcriptomic data between ’KDML105’ and CSSL18 at two days after salt stress were analyzed. All the DEGs were then mapped against the *Oryza sativa* Japonica group “Osa_MSU_v7.m02” database in MapMan. A regulation overview based on transcription factors, receptor kinases, phosphoinositides, and calcium regulation revealed 34 genes that were enriched in this analysis (Table S6). Interestingly, seven genes located on the introgress region on chromosome 1 were enriched (Table 1). Two of them, *LOC_Os01g72370* and *LOC_Os01g72530*, were selected for further characterization using the ortholog Arabidopsis mutant lines.

### 3.6. Physiological Trait Analysis in Arabidopsis Mutant Lines

**Figure 9:**
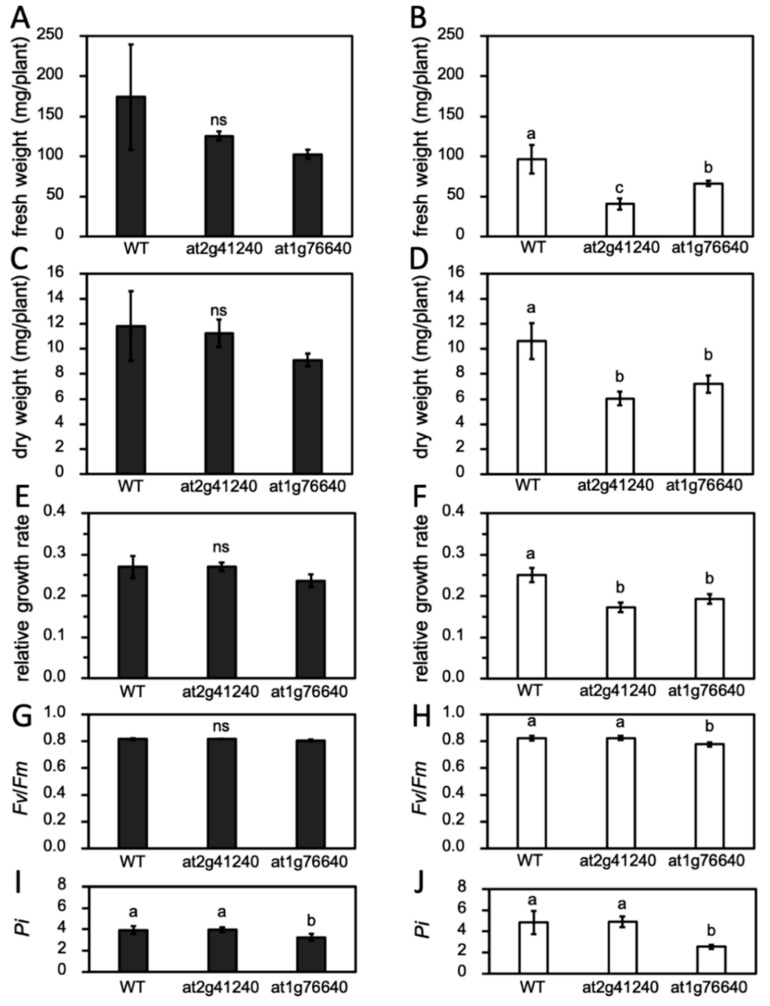
Phenotype of the *Arabidopsis* wild type (WT) and mutant under normal and salt stress conditions (250 mM NaCl) at 7 days after treatment in saline soil. Fresh weight (**A**,**B**), dry weight (**C**,**D**), relative growth rate (**E**,**F**), PSII maximum efficiency (*F_v_*/*F_m_*) (**G**,**H**), and performance index (*P_i_*) (**I**,**J**) of *Arabidopsis* WT, *at2g41240*, and *at1g76640* mutants under normal (represented with black bars) and 250 mM NaCl salt stress condition (represented with white bars). Different letters represent significant differences between groups (*p* < 0.05) and ns means no significant difference among means. Vertical bars represent standard deviation.

In the normal growth condition, both mutant lines were slightly smaller than the wild type (WT), but the fresh weight (Figure 9A) and dry weight (Figure 9C) of all lines were not significantly different. However, under the salt stress condition, the fresh weight and dry weight of all lines were decreased and resulted in the fresh weight and dry weight of the mutant lines being significantly lower than those of the WT (Figure 9B,C). RGR of all lines were similar when grown in the normal condition (Figure 9E). Under salt stress, both mutants had a lower RGR than the WT (Figure 9F).

Further investigation was performed with the measurement of PSII maximum efficiency (*F_v_*/*F_m_*) (Figure 9G,H) and *P_i_* (Figure 9I,J) because the reduction of the growth rate may have resulted in an impaired photosynthesis apparatus due to salt stress. All plants under the normal condition had a *F_v_*/*F_m_* value range of 0.81–0.84, indicating appropriate plant growth (Figure 9G). Under salt stress for seven days, both the WT and *at2g1240* maintained *F_v_*/*F_m_* (Figure 9H) and P_i_ (Figure 9I). Only the *at1g76640* mutant had a significantly lower *F_v_*/*F_m_* and *P_i_*, suggesting the higher PSII sensitivity to salt stress of this mutant line.

## 4. Discussion

### 4.1. Salt Tolerance Performance of the Four CSSLs

A number of CSSLs with different segments of chromosome 1 of DH212 in the ‘KDML105’ background were evaluated for their salt tolerance. CSSL18, which contains the full segment in the salt-tolerant QTL region from chromosome 1, had the best salt tolerance phenotype; that is, higher leaf water potential, root fresh weight, and higher RGR (7–14 day after stress) than the other CSSLs tested. CSSL11, which also contains the full segment of this QTL region based on simple sequence repeat (SSR) markers, did not show a similar phenotype. This suggested that other regions in the genome also contributed to the salt stress response. However, it cannot be ruled out that the SSR markers did not cover every locus in the salt tolerant QTL region reported by [5].

CSSL14 had the second best salt stress phenotype and CSSL22 had the worst salt stress phenotype. According to the SSR mapping of these two lines, the region between RM3468 and RM3362 may contain important loci for salt tolerance, whereas the region between RM3285 and RM3498 may not. However, a line like CSSL18 that contains the full segment may perform better under salt stress, although it is possible that important loci in regions that were not mapped with the SSR markers could exist in CSSL18.

### 4.2. Transcriptome Analysis for Salt Responses Reveals Salt Tolerance Candidate Genes

Significantly, DEGs between ‘KDML105’ and CSSL18 were found on all chromosomes, supporting the idea that multiple gene regulation and various mechanisms are required for salt stress responses [18,19].

In this study, we showed that gene enrichment analysis helps to determine the mechanism involved and to detect candidate genes for salt tolerance, similar to what was shown in other studies [20,21]. The GO enrichment analysis revealed that the term “microtubule-based process” was significantly enriched only in CSSL18. In *Arabidopsis*, the rapid depolymerization of microtubules and the formation of new microtubule networks contribute to survival under salt stress [22]. Moreover, OsDREPP2, a Ca^2+^ binding protein that also binds to microtubules, was shown to disappear after salt stress and then recover in ‘Pokkali’ rice, the salt-tolerant cultivar, but it did not recover in IR29, the salt-susceptible cultivar [23]. These data suggest that a microtubule-based process may contribute to CSSL18 salt stress adaptation.

The GO enrichment analysis revealed signal transduction differences in ‘KDML105’ and CSSL18, and two loci, *LOC_Os04g23890*, encoding an ACG kinase, and *LOC_Os07g44330*, encoding PDK, were detected. These two loci are not located in the salt-tolerant QTL region on chromosome 1. However, it may be that the expression of these two loci is regulated epistatically by genes in the QTL region, but that the expression, mechanism of action, or genetic nature of these regulatory loci prevents their detection.

The orthologous gene of *LOC_Os04g23890* in *Arabidopsis* encodes phototropin 2, which acts as a signal transducer in the phototropic response and in the stomatal opening [24]. It also mediates the transient increase of cytosolic Ca^2+^ in leaves [25]. Proteomic analysis of *Physcomitrella patens* under salt stress showed that phototropin was one of the signaling proteins that might regulate plasma membrane H^+^ ATPase and maintain ion homeostasis during salt stress [26]. The expression of rice phototropin 2 (*OsPHOT2*) was shown to be induced by white, blue, red, and far-red light [27]. Phototropin was also shown to be involved in chloroplast movement and was required for chloroplast gene expression [28]. While there is no published evidence of the involvement of *OsPHOT* in the salt stress response, our results suggest that *OsPHOT2* may contribute to the salt-tolerant phenotype of CSSL18. We found that *OsPHOT2* expression was maintained under salt stress, implying a role of *OsPHOT2* in ion homeostasis, similar to what was found in *Physcomitrella patens*.

PDK regulates the activity of pyruvate dehydrogenase, which catalyzes the conversion of pyruvate to acetyl-CoA. Upregulation of pyruvate dehydrogenase activity leads to enhancement of the energy producing pathway. PDK regulates pyruvate dehydrogenase by phosphorylating the E1 subunit in the pyruvate dehydrogenase complex and leads to the inhibition of pyruvate dehydrogenase activity [29,30]. We found that *PDK* was upregulated in ‘KDML105’ but showed no response to salt stress in CSSL18 when compared with the controls grown under normal conditions. This implies that pyruvate dehydrogenase activity was inhibited more in ‘KDML105’ than in CSSL18, leading to downregulation of the energy producing pathway, and indicates higher energy productivity in CSSL18 than in ‘KDML105’.

Enhancement of energy production was suggested to be important in salt tolerance mechanisms. For example, comparison of the rice root proteomes of salt-tolerant and salt-susceptible cultivars showed that proteins that function in the energy producing pathway were more abundant in the salt-tolerant cultivar [31]. In cucumber treated with exogenous putrescine, increased activity of the energy producing pathway was proposed to be the mechanism used to defend against salt injury, leading to salt tolerance [32]. In our study, salt stress induced higher expression of *PDK* in ‘KDML105’, but not in CSSL18. This indicates that CSSL18 had enhanced energy production compared with ‘KDML105’, leading to its better salt-tolerant phenotype.

### 4.3. Regulation Overview in MapMan Revealed Crucial Elements of the Signaling Pathway for Salt Stress Response in CSSL18

MapMan is a tool that permits high-throughput visualization of omics data by using a hierarchical BIN-based ontology system [33]. This study revealed that seven genes located on the introgress region on chromosome 1 were enriched. Some were reported to be involved in abiotic stress responses. Therefore, we selected two genes for characterization of the role in salt stress by using the *Arabidopsis* mutants that contained an insertion at the gene orthologous to the gene of interest in rice. These two genes were *OsIRO2* (iron-related transcription factor 2, *LOC_OS01g72370*) and *OsMSR2* (*multi*-*stress*-*responsive gene 2*, *LOC_Os01g72530*).

*OsIRO2* encodes the bHLH transcription factor responsible for regulation of the genes involved in iron homeostasis in rice and growth and yield improvement in calcareous soil [34]. The orthologous gene of *OsIRO2* in *Arabidopsis* is *At2g41240*, which encodes basic helix–loop–helix 100 (bHLH100). It was found to respond to selenium stress in Arabidopsis [35]. Moreover, the *at2g41240* mutant also exhibited increased hypocotyl elongation relative to the WT under a short day length condition. Recent studies revealed that *At2g41240* was proposed to be expressed under drought stress [36]. In our study, the *at2g41240* mutant showed growth inhibition under salt stress. This implied that *At2g41240* plays a role in the salt stress response.

*OsMSR2* (multi-stress-responsive gene 2, *LOC_Os01g72530*) is another gene that was reported to be involved in abiotic stress response. It modulated salt and drought tolerance in *Arabidopsis* through ABA-mediated pathways and increased ABA sensitivity in *Arabidopsis* [37]. Its orthologous gene in *Arabidopsis* is *At1g76640,* encoding calmodulin like 39 (*CML39)* related to *CML38*, which is a tandem gene with 76% amino acid sequence identity. Lokdarshi et al. [38] studied the transcriptional levels of *CML38* and *CML39* in Arabidopsis seedlings under oxygen deficiency stress. They reported that *CML38* showed an abundance of transcript levels over 300-fold in roots and approximately 100-fold in the shoots, at 6 hours after stress. However, *CML39* showed only a minor (five-fold) increase, indicating that *CML39* is less important under oxygen deficiency stress. Midhat et al. [39] reported that *CML39* was essential in various processes of development, from seeds to mature *Arabidopsis* plants, and acted as a stress-induced gene, which highlighted the importance of calcium signaling in plant development. In this study, the mutation on *At1g76640* not only resulted in growth inhibition under stress, but also decreased PSII maximum efficiency during salt stress. Chlorophyll fluorescence (*F_v_*/*F_m_)* has been used as an indicator of plant stress. Normally, an *F_v_*/*F_m_* value range of 0.79 to 0.84 is the optimal value for many plant species and lower values represent plant stress [40,41]. The photosynthetic performance index (*P_i_*) is the product of an antenna, reaction center, and electron transport dependent parameter [42]. *P_i_* is a very sensitive parameter in different crops and in stress conditions [42,43]. Moreover, Van Heerden et al. [44] observed a very good positive correlation between CO_2_ assimilation capacity and *P_i_* values under water stress. In our study, the *P_i_* maintenance ability in the *at11g76640* mutant declined after seven days of salt stress, indicating that plants were unable to control light harvest and photosynthetic capacities under salt stress. This suggested that this gene has a role in photosynthesis adaptation during salt stress.

## 5. Conclusions

We compared chromosome segment substitution lines (CSSLs) with the ‘KDML105’ genetic background for their salt tolerance and found that CSSL18 had the highest salt tolerance among the CSSLs tested. Comparison of the transcriptome pathways of CSSL18 and ‘KDML105’revealed *OsPHOT2* and *OsPDK* as candidate genes involved in the salt tolerance mechanism. Quantitative RT-PCR validation supported the role of these two genes in salt tolerance mechanisms. Moreover, the enrichment of two genes encoding the transcription factors and signaling proteins in the QTL of chromosome 1, *OsIRO2* and *OsMSR2*, showed the potential to be involved in salt stress response, especially, *OsMSR2* or *OsCML31*, whose orthologous genes in *Arabidopsis* had the potential role in photosynthesis adaptation under salt stress.

## Figures and Tables

**Table 1:** Regulatory genes located on the salt tolerance QTL from chromosome 1 enriched from MapMan analysis compared between ’KDML105’ and CSSL18 after 2 days of salt stress.

Locus	Bin Code	Bin Name	Description	Log2 Fold Changed	*A. thaliana* Ortholog	*A. thaliana* Mutant Stock
*LOC_Os01g53920*	30.2.11	signaling.receptor kinases.leucine rich repeat XI	Receptor-like protein kinase 5 precursor	1.07	-	-
*LOC_Os01g61080*	27.3.32	RNA.regulation of transcription.WRKY domain transcription factor family	WRKY24	0.89	*At2g38470*	SALK_006603
*LOC_Os01g65370*	27.3.25	RNA.regulation of transcription.MYB domain transcription factor family	MYB family transcription factor	1.04	-	-
*LOC_Os01g65650*	30.2.11	RNA.regulation of transcription.WRKY domain transcription factor family	Receptor-like protein kinase	1.04	*At1g72180*	SALK_081193C
*LOC_Os01g72100*	30.3	signaling.calcium	OSCML10	0.66	*At1g24620*	SALK_007994
*LOC_Os01g72370*	27.3.6	RNA.regulation of transcription.bHLH, Basic Helix-Loop-Helix family	HLH DNA-binding domain containing protein/OsIRO2	0.80	*At2g41240*	SALK_074568C
*LOC_Os01g72530*	30.3	signaling.calcium	OsCML31/OsMSR2	0.97	*At1g76640*	SALK_078400C

## Supplementary Materials

The following are available online at https://www.mdpi.com/2073-4425/10/10/742/s1, Figure S1: Leaf water potential of the first fully expanded leaves of four CSSLs, parental lines ‘(KDML105’ and DH212), and salt-tolerant (‘Pokkali’) and salt-susceptible (IR29) lines grown under normal conditions (A), and after 75 mM NaCl (B), and 150 mM NaCl (C) salt treatment for 0–12 days, Table S1: Details of the loci of differentially expressed genes detected by analysis of the ‘KDML105’ transcriptomes under normal and salt stress conditions, Table S2: Details of the loci of differentially expressed genes detected by analysis of the CSSL18 transcriptomes under normal and salt stress conditions, Table S3: Description and GO annotations of the differentially expressed genes in both ‘KDML105’ and CSSL18 under salt stress, Table S4: Details of the genes that were differentially expressed only in ‘KDML105’ under salt stress, Table S5: Details of the genes that were differentially expressed only in CSSL18 under salt stress, Table S6: Regulatory genes enriched from MapMan analysis compared between ‘KDML105’ and CSSL18 after two days of salt stress.

## References

[1] Chinnusamy V., Schumaker K., Zhu J.K. (2004). Molecular genetic perspectives on cross-talk and specificity in abiotic stress signalling in plants. J. Exp. Bot..

[2] Hao W., Lin H.X. (2010). Toward understanding genetic mechanisms of complex traits in rice. J. Genet. Genom..

[3] Knight H., Knight M.R. (2001). Abiotic stress signalling pathways: Specificity and cross-talk. Trends Plant Sci..

[4] Zhu J.K. (2001). Plant salt tolerance. Trends Plant Sci..

[5] Kanjoo V., Jearakongman S., Punyawaew K., Siangliw J.L., Siangliw M., Vanavichit A., Toojinda T. (2012). Co-location of quantitative trait loci for drought and salinity tolerance in rice. Thai J. Genet..

[6] Lanceras J.C., Pantuwan G., Jongdee B., Toojinda T. (2004). Quantitative trait loci associated with drought tolerance at reproductive stage in rice. Plant Physiol..

[7] International Rice Research Institute (2002). Standard Evaluation System for Rice.

[8] Missirian V., Comai L., Filkov V. (2011). Statistical mutation calling from sequenced overlapping DNA pools in TILLING experiments. BMC Bioinform..

[9] Langmead B., Salzberg S.L. (2012). Fast gapped-read alignment with Bowtie 2. Nat. Methods.

[10] Trapnell C., Pachter L., Salzberg S.L. (2009). TopHat: Discovering splice junctions with RNA-Seq. Bioinformatics.

[11] Anders S., Huber W. (2010). Differential expression analysis for sequence count data. Genome Biol..

[12] Maere S., Heymans K., Kuiper M. (2015). BiNGO: A Cytoscape plugin to assess overrepresentation of gene ontology categories in biological networks. Bioinformatics.

[13] Usadel B., Poree F., Nagel A., Lohse M., Czedik-Eysenberg A., Stitt M. (2009). A guide to using MapMan to visualize and compare Omics data in plants: A case study in the crop species, Maize. Plant Cell Environ..

[14] Pfaffl M.W. (2001). A new mathematical model for relative quantification in real-time RT-PCR. Nucleic Acids Res..

[15] Alonso J.M., Stepanova A.N., Leisse T.J., Kim C.J., Chen H., Shinn P., Stevenson D.K., Zimmerman J., Barajas P., Cheuk R. (2003). Genome-wide insertional mutagenesis of *Arabidopsis thaliana*. Science.

[16] Murashige T., Skoog F. (1962). A revised medium for rapid growth and bio assays with tobacco tissue cultures. Physiol. Plant..

[17] Ouyang S., Zhu W., Hamilton J., Lin H., Campbell M., Childs K., Thibaud-Nissen F., Malek R.L., Lee Y., Zheng L. (2007). The TIGR Rice Genome Annotation Resource: Improvements and new features. Nucleic Acids Res..

[18] Ali A., Yun D.J. (2017). Salt stress tolerance; what do we learn from halophytes?. J. Plant Biol..

[19] Yu J., Zao W., He Q., Kim T.S., Park Y.J. (2017). Genome-wide association study and gene set analysis for understanding candidate genes involved in salt tolerance at the rice seedling stage. Mol. Genet. Genom..

[20] Wu Q., Bai X., Zhao W., Xiang D., Wan Y., Yan J., Zou L., Zhao G. (2017). *De novo* assembly and analysis of tartary buckwheat (*Fagopyrum tataricum* Garetn.) transcriptome discloses key regulators involved in salt-stress response. Genes.

[21] Xiong H., Guo H., Xie Y., Zhao L., Gu J., Zhao S., Li J., Liu L. (2017). RNAseq analysis reveals pathways and candidate genes associated with salinity tolerance in a spaceflight-induced wheat mutant. Sci. Rep..

[22] Wang S., Kurepa J., Hashimoto T., Smalle J.A. (2011). Salt stress–induced disassembly of *Arabidopsis* cortical microtubule arrays involves 26S proteasome–dependent degradation of SPIRAL1. Plant Cell.

[23] Yamada N., Theerawitaya C., Kageyama H., Cha-Um S., Takabe T. (2015). Expression of developmentally regulated plasma membrane polypeptide (DREPP2) in rice root tip and interaction with Ca^2+^/CaM complex and microtubule. Protoplasma.

[24] Inada S., Ohgishi M., Mayama T., Okada K., Sakai T. (2004). RPT2 is a signal transducer involved in phototropic response and stomatal opening by association with phototropin 1 in *Arabidopsis thaliana*. Plant Cell.

[25] Harada A., Sakai T., Okada K. (2003). Phot1 and phot2 mediate blue light-induced transient increases in cytosolic Ca^2+^ differently in *Arabidopsis* leaves. Proc. Natl. Acad. Sci. USA.

[26] Wang X., Yang P., Gao Q., Liu X., Kuang T., Shen S., He Y. (2008). Proteomic analysis of the response to high-salinity stress in *Physcomitrella patens*. Planta.

[27] Jain M., Sharma P., Tyagi S.B., Tyagi A.K., Khurana J.P. (2007). Light regulation and differential tissue-specific expression of phototropin homologues from rice (*Oryza sativa* ssp. *indica*). Plant Sci..

[28] Goh C.H. (2009). Phototropins and chloroplast activity in plant blue light signaling. Plant Signal. Behav..

[29] Harris R.A., Huang B., Wu P. (2001). Control of pyruvate dehydrogenase kinase gene expression. Adv. Enzym. Regul..

[30] Sugden M.C., Holness M.J. (2003). Recent advances in mechanisms regulating glucose oxidation at the level of the pyruvate dehydrogenase complex by PDKs. Am. J. Physiol. Endocrinol. Metab..

[31] Damaris R.N., Li M., Liu Y., Chen X., Murage H., Yang P. (2016). A proteomic analysis of salt stress response in seedlings of two African rice cultivars. Biochim. Biophys. Acta Proteins Proteom..

[32] Zhong M., Yuan Y., Shu S., Sun J., Guo S., Yuan R., Tang Y. (2016). Effects of exogenous putrescine on glycolysis and Krebs cycle metabolism in cucumber leaves subjected to salt stress. Plant Growth Regul..

[33] Jung M., Jun H.B., Kim K.W., Suh H.W. (2010). Ontology mapping-based search with multidimensional similarity and Bayesian network. Int. J. Adv. Manuf. Technol..

[34] Ogo Y., Itai R.N., Kobayashi T., Aung M.S., Nakanishi H., Nishizawa N.K. (2011). OsIRO2 is responsible for iron utilization in rice and improves growth and yield in calcareous soil. Plant Mol. Biol..

[35] Van Hoewyk D., Takahashi H., Inoue E., Hess A., Tamaoki M., Pilon-Smits E.A. (2008). Transcriptome analyses give insights into selenium-stress responses and selenium tolerance mechanisms in Arabidopsis. Physiol. Plant..

[36] Rasheed S., Bashir K., Matsui A., Tanaka M., Seki M. (2016). Transcriptomic analysis of soil-grown *Arabidopsis thaliana* roots and shoots in response to a drought stress. Front. Plant Sci..

[37] Xu G.Y., Rocha P.S., Wang M.L., Xu M.L., Cui Y.C., Li L.Y., Zhu Y.X., Xia X. (2011). A novel rice calmodulin-like gene, *OsMSR2*, enhances drought and salt tolerance and increases ABA sensitivity in Arabidopsis. Planta.

[38] Lokdarshi A., Conner W.C., McClintock C., Li T., Roberts D.M. (2016). Arabidopsis CML38, a calcium sensor that localizes to ribonucleoprotein complexes under hypoxia stress. Plant Physiol..

[39] Midhat U., Ting M.K., Teresinski H.J., Snedden W.A. (2018). The calmodulin-like protein, cml39, is involved in regulating seed development, germination, and fruit development in Arabidopsis. Plant Mol. Biol..

[40] Kitajima M., Butler W. (1975). Quenching of chlorophyll fluorescence and primary photochemistry in chloroplasts by dibromothymoquinone. BBA-Bioenergetics.

[41] Maxwell K., Johnson G.N. (2000). Chlorophyll fluorescence—A practical guide. J. Exp. Bot..

[42] Oukarroum A., El Madidi S., Schansker G., Strasser R.J. (2007). Probing the responses of barley cultivars (*Hordeum vulgare* L.) by chlorophyll a fluorescence OLKJIP under drought stress and re-watering. Environ. Exp. Bot..

[43] Strasser R.J., Srivastava A., Tsimilli-Michael M., Yunus M., Pathre U., Mohanty P. (2000). The fluorescence transient as a tool to characterize and screen photosynthetic samples. Probing Photosynthesis: Mechanisms, Regulation and Adaptation.

[44] Van Heerden P., Swanepoel J., Krüger G. (2007). Modulation of photosynthesis by drought in two desert scrub species exhibiting C_3_-mode CO_2_ assimilation. Environ. Exp. Bot..

