# Supplementary figures and images for: Comparison between the Transcriptomes of ‘KDML105’ Rice and a Salt-Tolerant Chromosome Segment Substitution Line
Source: Genes (Basel). 2019 Sep 24;10(10):742. doi: 10.3390/genes10100742 (PMC6827086; doi:10.3390/genes10100742)

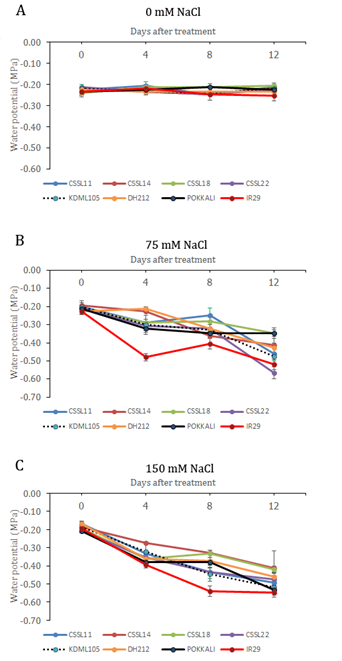

Supplement: Supplementary file 1 [file genes-10-00742-s001.zip › Supplementary/Supp Fig 1.tif]
